# Oral Squamous Cell Carcinoma of the Right Buccal Mucosa: A Case Report

**DOI:** 10.7759/cureus.59571

**Published:** 2024-05-03

**Authors:** Ponnusamy Subramani Gayathri, Bhuvaneshwari M, Priya Ramani, Manju J, Sreedevi Jeyakumaran, Praveena Raman

**Affiliations:** 1 Oral Medicine and Radiology, Thai Moogambigai Dental College and Hospital, Chennai, IND; 2 Oral Medicine and Radiology, Asan Memorial Dental College and Hospital, Chennai, IND

**Keywords:** carcinoma of buccal mucosa, scc (squamous cell carcinoma) buccal mucosa, oral squamous cell carcinoma, human oral squamous cell carcinoma (oscc), chronic non-healing ulcer, leukoplakia, burning sensation, squamous cell carcinoma, ulcer

## Abstract

Oral squamous cell carcinoma (OSCC) is indeed one of the most common types of oral cancer, typically affecting individuals over the age of 50. It primarily originates from the squamous epithelial cells lining the oral cavity. While it is relatively rare in individuals under 40 years old, it can still occur, albeit less frequently in that age group. Risk factors for developing OSCC include tobacco use (smoking or chewing), excessive alcohol consumption, chronic irritation (such as from poorly fitting dentures), human papillomavirus (HPV), infection, and certain dietary foods. Early detection and treatment are crucial for improving outcomes and reducing the mortality associated with this type of cancer. This report describes a case of OSCC, staged T2 N0 M0, involving the right buccal mucosa of a 51-year-old male patient. The patient reported intense pain in an ulcer on the right side of his cheek. This report focuses on the etiological factors and a brief literature review of squamous cell carcinoma.

## Introduction

The most common oral malignancy globally is oral squamous cell carcinoma (OSCC) [1]. Cultural habits and various risk factors, such as tobacco and alcohol consumption, contribute to the variability in its incidence worldwide.

Despite the availability of numerous therapeutic strategies, the five-year survival rate for OSCC remains relatively low, at around 50%. This underscores the urgency of early detection, as survival rates can significantly improve when the cancer is treated at earlier stages, potentially reaching up to 80% [2].

However, diagnosing OSCC early can be challenging due to its varied clinical presentation. While persistent ulcerated lesions are a common manifestation, there can be other clinical features as well, complicating early detection. Primary prevention efforts typically involve educating individuals about the risk factors associated with OSCC, such as tobacco and alcohol use. However, a lack of awareness about early lesions and delays in referring patients for specialized clinical evaluation and biopsy contribute to many OSCC cases being diagnosed at an advanced stage.

Therefore, there is a clear need for increased awareness among both healthcare professionals and the general population regarding the signs and symptoms of OSCC, as well as the importance of timely referral and biopsy for suspicious lesions. These efforts can lead to earlier diagnosis, improved treatment outcomes, and ultimately, higher survival rates for individuals affected by OSCC [3].

## Case presentation

A 51-year-old male patient reported to the Department of Oral Medicine and Radiology with the chief complaint of an ulcer on the right side of the cheek region for the past four months. The history of the presenting illness revealed that the patient initially developed a small ulcer on the right side of the inner cheek region four months ago, which was associated with an on-and-off burning sensation when consuming hot and spicy foods. The ulcer was initially small in size, which gradually increased to attain the present size. The patient was healthy, and his medical history was not contributory. The patient has been a smoker for the past 30 years (three to four packs of cigarettes per day) and has been consuming alcohol for the past 30 years. The patient gave no history of chewing tobacco or other parafunctional habits.

On extra-oral examination, it was noted that the patient had a symmetrical face with normal eyes, ears, and nose, competent lips, and no scars, sinuses, or tenderness in the maxillary sinus. On lymph node examination, a single ovoid-shaped lymph node was palpable on the left submandibular region, which measured approximately. 1 x 1.5 cm was non-tender on palpation, soft in consistency, and freely movable on all planes.

**Figure 1:**
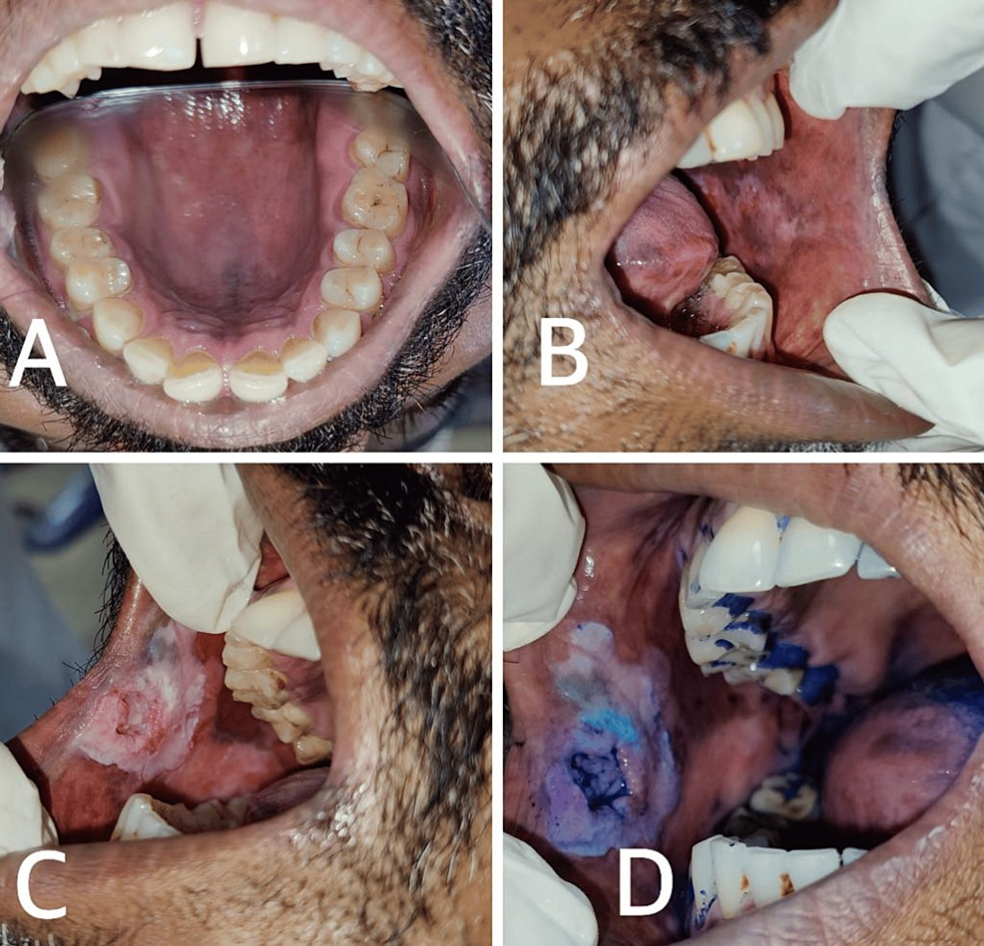
a) Diffused grayish-white area seen with small nodular excrescences having small central red spots all over the palate suggestive of smoker's palate; b) Greyish white patch with interspersed erythematous area seen in the left buccal mucosa; c) A single grayish-white diffused patch is seen with a single irregular erythematous large ulcer in the center. The edges appear to be raised, and the floor contains yellowish-white slough; d) Areas of dark blue indicate the uptake of toluidine staining in nuclei containing RNA and DNA. RNA: ribonucleic Acid; DNA: deoxyribonucleic acid

On intra-oral examination of the palate, a diffused grayish-white area was seen with small nodular excrescences having small central red spots all over the palate, extending anteriorly from the incisive papillae to posteriorly till the soft palate. No tenderness and no secondary changes were evident (Figure 1A).

On inspection of the left buccal mucosa, there was a greyish-white patch with an interspersed erythematous area seen extending anteriorly 5 mm away from the commissure of the lip to posteriorly till the retromolar region, superiorly from 2 mm from the plane of occlusion, and inferiorly till the lower buccal vestibule. On palpation, all the inspectory findings regarding the site, size, shape, extent, and number were confirmed. No tenderness and no secondary changes were evident, and it was smooth on palpation (Figure 1B).

On inspection of the right buccal mucosa, a single greyish-white diffused patch was seen extending from the commissure of the lip anteriorly to the occlusal plane tooth #45 posteriorly, which extended inferiorly 3 mm away from the lower buccal vestibule and superiorly 2 mm above the occlusal plane, roughly measuring about 3 x 3.5 cm in size, with a single irregular erythematous large ulcer in the center, the edges appeared to be raised, and the floor contained yellowish-white slough. On palpation, all inspectory findings regarding site, size, shape, extent, and number were confirmed; the patch was non-scrapable in nature. The ulcer was tender on palpation, with a raised and everted edge and an indurated base (Figure 1C).

As a chairside investigation, exfoliative cytology and toluidine blue staining were done (Figure 1D). The exfoliative cytology of the right buccal mucosa reported that an H&E-stained smear showed sheets of pleomorphic epithelial cells with an alteration in the nuclear-cytoplasmic ratio and hyperchromatic nuclei. Sheets of acute inflammatory cells were also seen. The exfoliative cytology of the left buccal mucosa reported that an H&E-stained smear showed desquamated cells without atypia, and few inflammatory cells were seen. The exfoliative cytology impression revealed type III cytology (intermediate) in the right buccal mucosa and class I cytology in the left buccal mucosa.

Based on the chief complaint and clinical examination, a provisional diagnosis of chronic non-healing ulcer of the right buccal mucosa was made, and other diagnoses were leukoplakia in the buccal mucosa, smoker’s melanosis in the right and left buccal mucosa, and smoker's palate.

The patient was further advised to undergo blood investigations and an incisional biopsy for a confirmatory diagnosis. The blood investigations revealed all parameters were within normal limits. An incisional biopsy was performed in the right and left buccal mucosa under all asepsis procedures with local anesthesia, and the specimen was submitted for histopathology.

The histopathology reported for A1 (right buccal mucosa) revealed that the given H&E-stained soft tissue section showed dysplastic ortho-keratotic stratified squamous epithelium in association with fibrous connective tissue. The dysplastic epithelial cells invaded the connective tissue stroma in the form of sheets and islands, showing dysplastic features like cellular and nuclear pleomorphism, altered nuclear-cytoplasmic ratio, abnormal mitoses, nuclear hyperchromatism, a few individual cell keratinizations, and keratin pear formation. The connective tissue exhibited diffuse chronic inflammatory cell infiltrates, blood vessels, and extravasated red blood cells (RBCs) suggestive of squamous cell carcinoma, well-differentiated in the right buccal mucosa.

The histopathology reported for B1 (left buccal mucosa) showed dysplastic ortho-keratotic stratified squamous epithelium in association with fibrous connective tissue. The surface epithelium showed dysplastic changes such as nuclear hyperchromatism, basilar hyperplasia, and bulbous rete ridges. These dysplastic changes were confined to the lower one-third of the epithelium. The connective tissue exhibited a focal area of chronic inflammatory cell infiltrate, adipocytes, melanin incontinence blood vessels, and extravasated RBCs, suggesting epithelial dysplasia, mild in the left buccal mucosa.

## Discussion

Oral squamous cell carcinoma is the predominant type of oral malignancy, accounting for approximately 95% of all cases [4,5]. It can manifest in various forms, including red, white, red-white, exophytic, or ulcerative lesions within the oral cavity. These lesions may be asymptomatic, leading to delayed detection as patients may not be aware of them until they have progressed significantly.

The most common occurrence of OSCC is in the buccal mucosa, which is 40%. The lower lip, floor of the mouth, and ventral and lateral borders of the tongue are the other commonly affected sites. While lesions are typically solitary, multifocal occurrences are rare [6].

Several factors increase the likelihood of OSCC, including being male, older than 40 years, heavy smoking or alcohol consumption, absence of trauma or systemic disease, negative serologic findings, and lesion localization not on the posterolateral region of the hard palate [7]. Oral leukoplakia was associated with a 40.8-fold increased risk of oral cancer and a five-year absolute risk of 3.3%. 

The classic presentation of OSCC in the buccal mucosa is characterized by a persistent, painful ulcer with induration and infiltration of deeper tissues in the oral cavity. In certain cases, they are superficial and appear to be growing outward rather than invading deeper into the tissues [7].

Early and accurate diagnosis of OSCC is crucial for improving patient prognosis and survival rates, as the ulcerative form of SCC is locally destructive. Treatment options for OSCC vary and may include surgery, radiotherapy, chemotherapy, or a combination of these modalities. Tailoring treatment to individual cases is essential for optimizing outcomes in patients with OSCC [5,8].

In our case report, a 51-year-old male patient initially presented with a small white patch, which gradually developed into a chronic, non-healing ulcer. He had a deleterious habit of smoking and alcohol consumption and no medical history; his blood investigation was also within normal limits. An immediate incisional biopsy was done, and the patient was diagnosed with squamous cell carcinoma. Further, we referred the patient to a cancer institute, where the patient underwent surgical excision followed by chemotherapy. The patient is currently stable after one year of surgical excision and chemotherapy.

## Conclusions

In conclusion, it is important to detect potentially malignant lesions and manage them properly in time to prevent them from turning into malignancies. However, raising awareness about the importance of regular oral checkups is also crucial for the detection and prevention of OSCC. The mortality rates of OSCC among the general population cannot completely vanish but can definitely be reduced by a few steps, such as lifestyle changes and broad educational programs.
